# COVID-19-Induced Myocarditis: Pathophysiological Roles of ACE2 and Toll-like Receptors

**DOI:** 10.3390/ijms24065374

**Published:** 2023-03-11

**Authors:** Patrizia Pannucci, Sophie R. Jefferson, Jonathan Hampshire, Samantha L. Cooper, Stephen J. Hill, Jeanette Woolard

**Affiliations:** 1 Division of Physiology, Pharmacology and Neuroscience, School of Life Sciences, University of Nottingham, Nottingham NG7 2UH, UK; 2 Centre of Membrane Proteins and Receptor, University of Nottingham, Nottingham NG7 2UH, UK; 3 School of Medicine, University of Nottingham, Nottingham NG7 2UH, UK; 4 School of Medicine, University of Oxford, Oxford OX3 9DU, UK

**Keywords:** COVID-19, SARS-CoV-2, myocarditis, angiotensin-converting enzyme 2 (ACE2), Toll-like receptors (TLRs), cardiovascular system

## Abstract

The clinical manifestations of the severe acute respiratory syndrome coronavirus 2 (SARS-CoV-2) infection responsible for coronavirus disease 2019 (COVID-19) commonly include dyspnoea and fatigue, and they primarily involve the lungs. However, extra-pulmonary organ dysfunctions, particularly affecting the cardiovascular system, have also been observed following COVID-19 infection. In this context, several cardiac complications have been reported, including hypertension, thromboembolism, arrythmia and heart failure, with myocardial injury and myocarditis being the most frequent. These secondary myocardial inflammatory responses appear to be associated with a poorer disease course and increased mortality in patients with severe COVID-19. In addition, numerous episodes of myocarditis have been reported as a complication of COVID-19 mRNA vaccinations, especially in young adult males. Changes in the cell surface expression of angiotensin-converting enzyme 2 (ACE2) and direct injury to cardiomyocytes resulting from exaggerated immune responses to COVID-19 are just some of the mechanisms that may explain the pathogenesis of COVID-19-induced myocarditis. Here, we review the pathophysiological mechanisms underlying myocarditis associated with COVID-19 infection, with a particular focus on the involvement of ACE2 and Toll-like receptors (TLRs).

## 1. Introduction

Characterised by infiltration of immunocompetent cells into the myocardium, cardiomyocyte degeneration and non-ischemic necrosis [1,2], myocarditis is clinically described as an inflammatory disease of the cardiac muscle, whose aetiology and histopathologic pattern are characterised by a vast heterogeneity [3]. This heterogeneity is also true of its clinical manifestations, which include chest pain, dyspnoea, arrythmias and acute coronary-syndrome-like symptoms [1]. Although myocarditis is usually self-limiting and normal cardiac function is often restored, in some cases, cardiogenic shock, chronic dilated cardiomyopathy, heart failure and sudden cardiac death (SCD) can occur [4]. Myocarditis-related SCD is often observed in young and active populations [5,6], and the consumption of performance enhancers has been reported to be one cause of myocardial inflammation in athletes [7]. 

While myocarditis continues to be underdiagnosed, its incidence is estimated to be between 10 and 20 cases per 100,000 individuals, with around 1.5 million cases reported worldwide annually [8,9]. The prevalence of myocardial inflammation varies according to ethnicities, gender and age, with young men showing a higher incidence rate [9,10].

Although both infectious and non-infectious agents can contribute to the aetiology of myocarditis, cardiotropic viral infections represent the most common cause [3]. Myocarditis is mainly attributable to enteroviruses, principally Coxsackievirus B3, but the list of viruses recognised as causative agents for myocardial inflammation has grown over the years and includes cytomegalovirus, adenoviruses, Parvovirus B19, Herpes Simplex viruses, Epstein-Barr virus, Hepatitis C virus, Influenza virus and Human Immunodeficiency Virus [11,12]. With the recent rapid spread of COVID-19, numerous cases of SARS-CoV-2-related myocarditis have now been described [13,14,15,16]. Severe acute respiratory syndrome coronavirus (SARS-CoV-1) and Middle East respiratory syndrome coronavirus (MERS-CoV) can also cause myocardial inflammation [17,18]. The pathogenetic mechanisms involved are similar to those previously described for Influenza viruses, leading to an autoimmune response and a cytokine storm [19]. Belonging to the same *Coronaviridae* family as MERS-CoV and SARS-CoV-1, SARS-CoV-2 has been suggested to cause inflammatory cardiomyopathy through similar mechanisms [18,19,20]. 

The exact pathophysiological aspects underlying COVID-19-induced myocarditis are, so far, not clearly characterised, and several additional hypotheses have been suggested, including direct myocardial injury following the interaction between SARS-CoV-2 and ACE2, downregulation of ACE2 and consequent overstimulation of the renin–angiotensin–aldosterone system (RAAS) [14,21]. Furthermore, hyperinflammatory status due to an overactivation of TLRs, T-cell mediated myocardial cytotoxicity and exaggerated cytokine production could also have a role in COVID-19-induced myocarditis [14,21,22,23]. Multiple episodes of myocarditis have also been observed following mRNA COVID-19 vaccination, especially in young men [24,25]. Molecular mimicry, exaggerated immune response to mRNA and dysregulation of the immune system are some of the mechanisms proposed to explain the development of myocarditis following mRNA COVID-19 vaccination [24,26]. 

Here, we reviewed the proposed pathogenetic mechanisms (e.g., direct virus-induced myocardial toxicity, hyperimmune events and exaggerated inflammatory response) involved in the development of myocardial injury after SARS-CoV-2 infection, with a specific focus on: (1) the role of cardiovascular factors, such as ACE2 and other elements of RAAS, and (2) components of the immune system, specifically TLRs. ACE2 is important because it serves both as a cell surface receptor for SARS-CoV-2 entry as well as playing a crucial role within the cardiovascular system, where it acts as a regulatory component of the RAAS, resulting in the fine control of blood pressure homeostasis and fluid-electrolyte balance [27,28,29]. TLRs are key receptors in the innate immune response to pathogens [30]. In addition to being expressed on immune cells, they are also expressed by vascular endothelial cells, cardiac fibroblasts, vascular smooth muscle cells and cardiomyocytes [31,32,33]. As a result, dysregulated TLR signalling has been associated with the development of cardiovascular diseases, such as atherosclerosis, congestive heart failure, ischaemia-reperfusion injury and viral myocarditis [31,33,34].

## 2. ACE2 Biology and Physiological Functions

**Figure 1:**
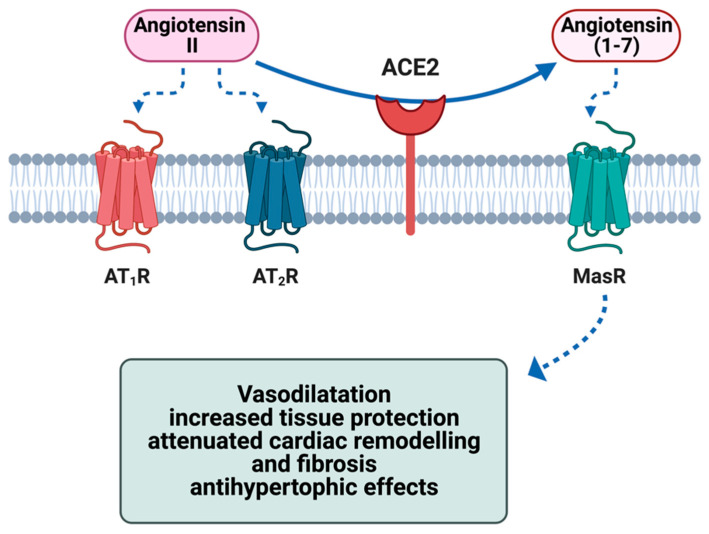
Effect of ACE2 on the metabolism of angiotensin II and the cytoprotective effects of the metabolite angiotensin (1-7) in the heart via activation of the G-protein-coupled receptor MasR. ACE2 = angiotensin-conversing enzyme 2; AT_1_R = angiotensin II type 1 receptor; AT_2_R = angiotensin II type 2 receptor; MasR = Mas receptor Figure created with Biorender.

ACE2 has been recently reported to have a pivotal role in the pathogenesis of SARS-CoV-2-induced myocardial inflammation [35]. This transmembrane glycoprotein is widely expressed in the respiratory system but has also been detected in cardiomyocytes, endothelial cells, vascular smooth muscle cells and cardiac fibroblasts [36]. ACE2 is a monocarboxypeptidase whose catalytic site promotes the conversion of angiotensin II (Ang II), a prohypertensive and profibrotic peptide, into angiotensin (1-7) (Ang (1-7)), a vasodilatory and cardioprotective agent [37]. It, therefore, plays a pivotal role in the regulation of the RAAS [38]. A reduction in blood pressure or fluid homeostasis alterations initiate the activation of the RAAS via the release of renin from its inactive precursor in the juxtaglomerular apparatus [39,40]. The secretion of renin in the blood determines the conversion of angiotensinogen into angiotensin I (Ang I), which represents the inactive precursor of Ang II [29,39]. Angiotensin-converting enzyme (ACE), a carboxypeptidase mainly expressed in pulmonary and renal vascular endothelium, is then responsible for the conversion of Ang I into Ang II, interacting with angiotensin II type 1 (AT_1_) and type 2 (AT_2_) receptors; this mechanism is the major effector of the RAAS and produces vasoconstriction and a consequent increase in blood pressure [39,40]. ACE2 promotes the cleavage of Ang I and Ang II into angiotensin (1-9) (Ang (1-9)) and Ang (1-7), respectively [39]. By interacting with the Mas receptor (MasR), both these peptides exert vasoprotective and antiproliferative effects through the stimulation of nitric oxide production via Akt-dependent pathways, resulting in a fine endogenous counter-regulatory mechanism within the RAAS [41,42]. ACE2-mediated hydrolysis of Ang II to Ang-(1-7) and the activation of the ACE2/Ang II/Mas receptor axis antagonise the detrimental effects of ACE/Ang II/AT_1_ receptor axis, with beneficial outcomes on cardiovascular physiology and homeostasis [36,43]. Specifically, the ACE2/Ang II/Mas receptor axis-mediated cardioprotective effects include maintenance of endothelial function, suppression of reactive oxygen species (ROS) generation, enhanced atherosclerotic plaque stability, vasodilation, reduced salt and water retention, as well as inhibition of profibrotic and prohypertrophic mechanisms [44,45,46,47] (Figure 1). 

## 3. Toll-like Receptors: Biology and Physiological Role 

As a key component in innate and adaptive immune responses, TLRs have been recognised as pivotal elements in the pathogenesis of virus-induced myocarditis [33,48]. Belonging to the family of pattern recognition receptors, they respond both to damage- and pathogen-associated molecular patterns (DAMPs and PAMPs). PAMPs, including viral single-stranded (ss) RNA, double-stranded (ds) RNA, viral or bacterial DNA, lipopeptides, lipopolysaccharide and bacterial flagellin, represent the exogenous ligands of TLRs [49]. Molecules secreted or exposed by damaged tissues and dead cells (e.g., extracellular matrix components, cytosolic and nuclear proteins, plasma membrane components, heat-shock proteins) are defined as DAMPs and act as endogenous ligands for TLRs [50]. By recognizing PAMPs and endogenous pathogenic ligands, TLRs have a crucial role in the innate and adaptive immune responses [51]. 

TLRs consist of an N-terminal domain (NTD), which serves as binding site for TLR ligands, a transmembrane domain and a C-terminal domain (CTD), which is responsible for the interaction with adaptor molecules and the consequent activation of intracellular downstream signalling cascades [51]. The intracellular region of these receptors is characterised by the presence of the Toll/IL-1 receptor homologous domain (TIR), which, upon binding with adaptor molecules, initiates the signalling cascade within the cell [52,53]. In particular, the dimerised TIR domain on TLRs recognizes the TIR domain on distinct downstream adaptor molecules, which include MyD88 (myeloid differentiation primary-response protein 88), Mal (TIR domain-containing adaptor protein), TRIF (TIR domain-containing adaptor inducing IFN-β) and TRAM (TRIF-related adaptor molecule) [51,53]. Depending on the recruitment of either MyD88 or TRIF, two different intracellular pathways are activated, namely MyD88-dependent pathway (associated with an increased expression of proinflammatory cytokines) or TRIF-dependent pathway (culminating in an enhanced production of type I interferons) [51]. 

These transmembrane glycoprotein receptors, of which 10 subtypes have been identified (TLR1-10), are expressed not only on immune cells but have been detected also in the cardiovascular system [31]. In this context, protein expression of TLR2, TLR3, TLR4, TLR5, TLR7 and TLR9 has been confirmed in cardiomyocytes [31,54], where their activation has been shown to trigger myocardial recruitment of immune cells and the amplification of inflammatory response via the release of pro-inflammatory cytokines [12], thus suggesting a crucial involvement of their signalling pathways in the development of myocardial inflammation [33].

## 4. Virus-Induced Myocarditis and Its Pathophysiology

Although it is clear that viral myocarditis is the result of the interaction between a cardiotropic virus and the host immune system [1], the molecular pathogenetic events leading to the onset and progression of this disorder have not been clearly identified [5,55]. In this context, murine models have been proven to be an excellent experimental model to better characterise the pathophysiological mechanisms underlying virus-induced inflammatory cardiomyopathy [9,55]. Viral myocardial inflammation has been recognised as the result of three different phases: (1) an acute viral stage; (2) a subacute inflammatory stage; and (3) an immune-mediated phase. The final outcome may result in either resolution and subsequent recovery or chronic disease characterised by cardiac remodelling and cardiomyopathy [1,56]. 

Viral invasion of cardiomyocytes, resulting from a receptor-mediated endocytosis of viral pathogen, defines the first acute phase of myocarditis [8]. This event results in a direct destruction of cell structures and consequent cardiomyocyte damage, which triggers the activation of humoral and cell-mediated immune responses, which represent the second phase of the disease [56]. The immune response is crucial for the attenuation of viral replication, but it may also have a role in enhancing viral entry, especially through mechanisms, such as molecular mimicry, increased expression of immune-cell-adhesion molecules or co-receptors and activation of immune signalling pathways (e.g., p56^lck^, Fyn and Abl), which alter the host cell cytoskeleton and, therefore, facilitating virus internalization [1,56,57,58]. The second stage can lead either to the resolution of viral infection with subsequent recovery or to a third phase, which develops as a result of extensive damage to cardiac muscle [4,5].

This third phase is characterised by a subacute and then chronic inflammatory state that results in additional destruction of cardiomyocytes and pathological fibrosis, leading to myocardial remodelling and eventual progression to dilated cardiomyopathy (DCM) [55]. Although the exact pathogenetic events underpinning virus-induced myocardial inflammation are still controversial, three plausible mechanisms have been proposed: (1) a myocardial injury induced directly by the viral pathogen; (2) immune-mediated myocardial damage; or (3) autoimmune reactions leading to cardiomyocyte destruction [12]. 

Direct virus-induced damage of myocardial cells has been observed in several in vitro and in vivo studies, which indicate that viral entry and replication in cardiomyocytes directly triggers the apoptosis of these cardiac cells and subsequent cardiac remodelling and DCM [12,59]. Although cardiomyocyte apoptosis is also triggered by cytokines and cytotoxic elements (e.g., perforins and ROS) as a result of sustained inflammation, these studies discriminated the virus-induced from inflammation-induced cardiomyocyte apoptosis by evaluating the amount of viral proteins in apoptotic myocytes and blood, as well as measuring active caspase 3 activity and using terminal deozynucleotidyl transferase deoxyuridine, triphosphate (dUTP) nick-end labelling (TUNEL) assay [59]. At least in the early stage of virus-induced myocardial injury, when the cardiomyocyte apoptotic rate was already extensive, the presence of inflammatory components in the myocardium was negligible. This supports the hypothesis that the apoptosis of cardiac cells and consequent myocardial damage are the result of a direct virus-mediated cytotoxic insult [59,60]. In addition, some viral proteases have been demonstrated to cleave dystrophin, a cytoskeletal protein crucial for myocyte function, causing a direct myocardial injury and reinforcing the hypothesis of direct virus-induced damage of the cardiac muscle [12,61]. 

An exaggerated immune response leading to myocardial injury represents the second proposed mechanism [12]. After entry into the myocardium, viral particles are recognised by TLRs expressed on antigen-presenting cells, resulting in the activation of the transcription factor nuclear factor kappa B (NF-kB) and subsequent transcription of proinflammatory cytokines, such as interleukin-1 (IL-1), interleukin-6 (IL-6), interleukin-12 (IL-12), tumour necrosis factor alpha (TNFα) and interferon gamma (IFN-γ) [12]. Such cytokines determine the recruitment of immune cells, namely antigen-specific T cells (CD8^+^ and CD4^+^) and B cells, into the infected myocardium, with the aim of eradicating the viral pathogen [62]. However, a sustained release and infiltration of proinflammatory cytokines and immune cells into the myocardium may lead to cardiomyocyte damage and subsequent myocardial dysfunction [12,57,63,64].

Finally, myocardial injury can result from an autoimmune response against virus-infected cardiomyocytes, which may be provoked either by antigenic mimicry between viral and myocyte epitopes or by exposure of cardiac antigens resulting from cardiomyocyte destruction [65,66]. This autoimmune scenario is also supported by the presence of increased levels of cardiac autoantibodies in patients with myocarditis and DCM, in particular autoantibodies against cardiac actin and myosin heavy chain, laminin and β-adrenoceptors [67,68,69,70,71], which may explain cardiomyocyte injury and the resultant myocardial dysfunction [72]. 

## 5. Role of Cardiovascular Factors (ACE2 and RAAS) in the Pathogenesis of COVID-19-Related Myocarditis

Since the recent SARS-CoV-2 outbreak, numerous cases of myocarditis have been reported as associated with this viral pathogen [35,73]. In the period from March 2020 to January 2021, COVID-19 patients, on average, were at a 15.7-times increased risk of myocarditis than those without the disease [74]. In addition, this virus-induced cardiovascular manifestation has been associated with adverse outcomes and a poorer prognosis in COVID-19 patients [75]. Although the exact pathophysiological events underlining this cardiovascular complication are not well known, the pathogenesis of COVID-19-induced myocardial inflammation has been proposed to be due to a combination of a direct virally induced cardiomyocyte damage, an overactive immune response to infected cardiac muscle cells and an overproduction of proinflammatory cytokines [14,21]. The similarity in SARS-CoV-2 infection with those induced by other coronaviruses, namely MERS-CoV and SARS-CoV-1, whose viral RNAs were detected in both animal and human heart samples, suggests that even the new member of the *Coronaviridae* family may possess cardiotropism, meaning that cardiovascular tissues are susceptible to SARS-CoV-2 infection [14]; SARS-CoV-2 tropism for myocardial cells might be an important determinant of COVID-19-related myocarditis and the viral persistence in the myocardium [76].

**Figure 2:**
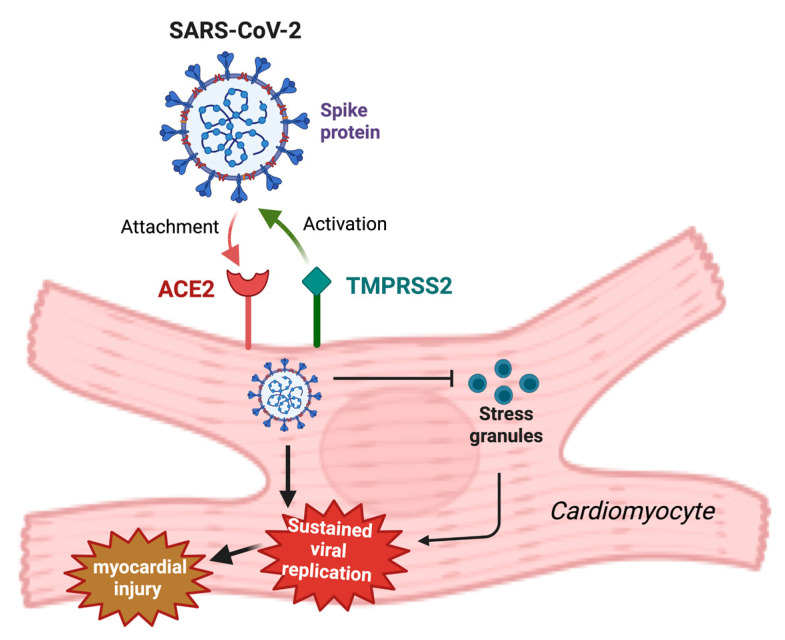
Proposed involvement of ACE2 in SARS-CoV-2-induced myocarditis. SARS-CoV-2 spike protein, after proteolytic activation by TMPRSS2, binds ACE2 on cardiomyocytes, enabling SARS-CoV-2 entry. Stress granule formation is then inhibited by intracellular SARS-CoV-2, leading to an increased viral replication and consequent amplified myocardial injury. ACE2 = angiotensin-converting enzyme; SARS-CoV-2 = severe acute respiratory syndrome coronavirus 2; TMPRSS2 = transmembrane serine protease 2. Created with BioRender.

Beyond its role as an antifibrotic, antihypertrophic and vasodilatory mediator of the cardiovascular peptide hormone system, ACE2 also acts as an entry receptor for SARS-CoV-2 [38]. As a result, this transmembrane protein has been suggested as a possible link between SARS-CoV-2-related immune response, inflammation and cardiovascular complications [77]. Indeed, SARS-CoV-2 viral RNA has been detected in autopsied hearts from infected patients [78]. In this context, the transmembrane serine protease 2 (TMPRSS2)-mediated cleavage of S1 subunit from SARS-CoV-2 spike protein promotes binding between the viral spike protein and ACE2, therefore, mediating SARS-CoV-2 internalisation [79] (Figure 2). SARS-CoV-2 spike protein is characterised by two subunits, namely S1 and S2 [80,81]. The proteolytic cleavage at the S1/S2 site by TMPRSS-2 is crucial for SARS-CoV-2 infection and allows for S1 that contains a receptor-binding domain (RBD) to bind to ACE2 on the host cell [81,82,83], whilst S2 initiates the fusion between viral and host cell membranes [81,84]. Once the virus has entered the cells, viral replication is promoted by SARS-CoV-2 nucleocapsid protein, which appears to have a role in inhibiting the formation of stress granules (SGs), non-membranous ribonucleoprotein assemblies, which, sequestering viral proteins, limit the replication of the virus [85]. As a result of impaired SG formation, SARS-CoV-2 evades antiviral host defence and the replication of its genome is then promoted [85]. 

TMPRSS2 is also responsible for the proteolysis of ACE2 [86,87]. ACE2 cleavage by TMPRSS2 has been suggested to further increase SARS-CoV-2 uptake, although the mechanism involved in the augmentation of viral entry mediated by TMPRSS2 is still unclear [86,87,88]. The formation of SARS-CoV-2/ACE2 complex permits virus to enter host cells via endocytosis, therefore, producing a downregulation in surface ACE2 and the consequent loss of its cardioprotective function, with profound effects on the cardiovascular system [43,77]. The resulting imbalance of RAAS in favour of ACE/Ang II/AT_1_ receptor axis promotes a sustained vasoconstrictive response, inflammation, hypertrophy, profibrotic status, as well as cardiac remodelling, leading to the myocardial impairment associated with SARS-CoV-2 infection [89]. 

SARS-CoV-2 also induces upregulation of a disintegrin and metalloprotease 17 (ADAM17), a protein belonging to the disintegrins and metalloproteinases family, which promotes a further reduction in membrane ACE2 expression by mediating its ectodomain shedding and the consequent release of a soluble form of ACE2 (sACE2), with a consequent accumulation of Ang II and overactivation of RAAS [89,90,91]. As a result, ADAM17-mediated release of the soluble form of ACE2 has been associated with an increased cardiovascular risk [77,92,93,94]. In addition, ADAM17-dependent proteolytic cleavage of ACE2 into a soluble form has also been found to have a role in further facilitating SARS-CoV-2 entry via AT_1_ receptors [95,96]. Indeed, it has been observed that sACE2, conserving the interaction site for SARS-CoV-2 spike protein, engages the virus, resulting in a complex that has been suggested to enter the cells via AT_1_ receptor-mediated endocytosis [95,97]. The same study revealed that sACE2 promotes SARS-CoV-2 cell entry by interacting also with vasopressin, therefore, forming an sACE2-viral spike protein–vasopressin complex, which elicits SARS-CoV-2 entry through binding with the arginine vasopressin receptor 1b (AVPR1B) receptor [95]. 

Disruption of the ACE2/Ang (1-7)/Mas receptor axis in COVID-19 patients, therefore, appears to be intimately involved in SARS-CoV-2-induced myocardial injury and this is supported by the decreased myocardial ACE2 expression detected in post-mortem heart samples [77,98].

## 6. Role of Immune System Components (Toll-like Receptors)

In addition to direct damage to the myocardium, SARS-CoV-2-induced myocarditis has been suggested to result from indirect effects on cardiac muscle induced by the immune system [99]. In this context, it is possible that antigen-presenting cells (APCs) process and deliver SARS-CoV-2 antigens to naïve T cells, which then become fully activated cytotoxic T cells (CD8^+^) [14]. However, at the present time, there is no direct evidence for this in COVID-19 patients. If this occurs, then heart-produced hepatocyte growth factor (HGF), interacting with the receptor tyrosine kinase c-MET expressed on naïve T lymphocytes, could induce T-cell cardiotropism and directly promote the recruitment of these activated T lymphocytes to the heart, where they would be able to evoke cell-mediated cytotoxicity and destruction of myocardial tissue [12,14,100]. Furthermore, the CD8^+^ cells that recognise SARS-CoV-2 antigen, processed and presented by major histocompatibility complex class I (MHC class I) on the surface of infected cardiomyocytes, may result in T-lymphocyte-mediated cytotoxicity and subsequent lysis of infected cardiomyocytes [12,14]. 

CD8^+^ cells are not only associated with a direct T-cell-mediated cytotoxic effect on myocardial cells but also with an amplified inflammatory response, known as a cytokine storm, which results in the overproduction of proinflammatory molecules, such as IL-6, IFN-γ and TNFα [14,101]. Such cytokines may indirectly cause myocardial dysfunction by promoting cardiomyocyte apoptosis, as well as the recruitment of immune cells to the infected myocardium [99,102]. The cytokines produced may also have a detrimental effect on ACE2 mRNA transcription, leading to decreased expression of ACE2, which, in turn, may be responsible for a deleterious increase in Ang II and loss of cardioprotection mediated by Ang (1-7)/mitochondrial assembly receptor (Ang (1-7)/MasR) axis [79] (Figure 1). 

In the context of exaggerated immune activity, a specific class of receptors within the innate immune system, namely TLRs (see below), may also play a crucial role in the pathogenesis of myocarditis [23]. Indeed, the activation of the TLR4 signalling pathway following the interaction between this receptor and SARS-CoV-2 spike protein has been proposed to increase the expression of surface ACE2, therefore, promoting viral entry and contributing to the pathogenetic mechanisms of SARS-CoV-2-induced myocarditis [23,103]. Aberrant TLR signalling also promotes a hyperinflammatory status and an exaggerated immune response that further contributes to the pathophysiological aspects of SARS-CoV-2-induced myocarditis [23,33].

**Figure 3:**
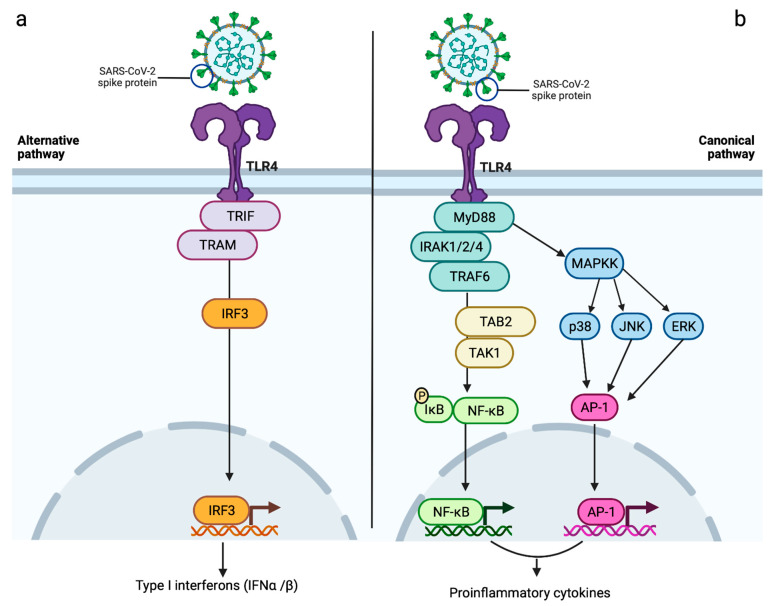
Proposed roles of TLR4 in SARS-CoV-2-induced myocarditis. Triggering a sustained immunoinflammatory response, this receptor may contribute to myocardial damage observed in COVID-19 patients via two different signalling pathways: (**a**) the interaction between SARS-CoV-2 and TLR4 determines the recruitment of TRIF and TRAM, resulting in the phosphorylation and consequent nuclear translocation of IRF3. This alternative pathway culminates in the production of type I interferons (IFNα and IFNβ), which might lead to an increased expression of surface ACE2 via ISGs, therefore, amplifying ACE2-induced myocardial damage; (**b**) the canonical pathway of TLR4, also known as MyD88-dependent pathway, promotes the recruitment and activation of IRAK 1/2/4, in turn, responsible for inducing the activation of TRAF6/TAB2/TAK1 cascade. The consequent phosphorylation-induced degradation of IκB and nuclear translocation of NF-kB results in the release of proinflammatory cytokines, which might have a role in SARS-CoV-2-induced myocarditis. As part of the TLR4 canonical pathway, the activation of MAPKK might also contribute to the increased transcription of proinflammatory cytokines via the phosphorylation of ERK, JNK and p38 and consequent nuclear translocation of AP-1. AP-1 = activator protein 1; ERK = extracellular signal-regulated kinase; IFNα = interferon alpha; IFNβ = interferon beta; IκB = inhibitor of nuclear factor kappa B; IRAK 1/2/4 = IL-1 receptor-associated kinases 1/2/4; IRF3 = interferon regulatory factor 3; ISGs = interferon-stimulated genes; JNK = c-Jun N-terminal kinase; MAPKK = mitogen-activated protein kinase kinase; NF-kB = nuclear factor kappa B; p38 = p38 mitogen-activated protein kinases; TAB2 = TGF-beta activated kinase 1 binding protein 2; TAK1 =transforming growth factor-β activated kinase 1; TRAM = TRIF-related adaptor molecule; TRAF6 = TNF receptor associated factor 6; TRIF = TIR-domain-containing adapter-inducing interferon β. Created with BioRender.

A role of TLR4 in SARS-CoV-2-induced myocarditis has been proposed since it binds with high affinity to the SARS-CoV-2 spike protein [23,104]. This might explain the hyperinflammatory status of cardiac muscle induced by SARS-CoV-2 as a result of both a prolonged immune response and an indirect effect on ACE2 surface expression [23]. The binding of SARS-CoV-2 spike protein to TLR4 results in the activation of an intracellular pathway characterised by the recruitment of the adapter molecules Toll/interleukin-1 receptor (TIR)-domain-containing adapter-inducing interferon β (TRIF) and TRIF-related adaptor molecule (TRAM), with the consequent production of type I interferons (IFNα and IFNβ), which are, in turn, responsible for promoting the expression of interferon-stimulated genes (ISGs) (Figure 3a) [23]. The TRIF-dependent signalling pathway is, indeed, associated with the activation of type I interferon response, crucial for its protective role in viral infection. Following SARS-CoV-2 infection, the interaction between viral particles and TLRs leads to the activation of the TRIF-dependent pathway and the consequent production of type I interferons, resulting in activation of the innate immune system [105,106,107]. Type I interferons bind the receptor complex consisting of interferon α and β receptor subunit 1 and 2 (IFNAR1/IFNAR2), leading to the activation of Janus kinase (JAK)-signal transducer and activator of transcription (STAT) pathway. This promotes the expression of ISGs [108], which play an important role in the inhibition of viral transcription and replication [106,108]. Although the TLR-mediated production of ISGs has a protective effect against SARS-CoV-2 [108], the ISGs resulting from activation of the TLR4 pathway have been proposed to increase surface ACE2 expression, therefore, amplifying the ACE2-mediated pathways of myocardial inflammation [23,109]. In this context, ACE2 has been suggested to act as an ISG; therefore, the type I interferon-induced expression of ACE2 may further aggravate SARS-CoV-2 manifestations, including myocarditis [109,110].

Serum levels of SARS-CoV-2 viral elements and/or DAMPs released by host-infected and necrotic cells detected in COVID-19 patients may also activate the TLR4 pathway via the myeloid differentiation factor 88 (MyD88)-dependent pathway, which, resulting in an amplified proinflammatory response, may also have a role in SARS-CoV-2-induced myocarditis [23,106,111,112,113]. TLR4-promoted recruitment of MyD88 leads to the activation of IL-1 receptor-associated kinases (IRAK-1, -2 and -4), which are, in turn, responsible for the degradation of the inhibitory protein IκB (inhibitor of nuclear factor kappa B) via TRAF6/TAK1/TAB2 (TNF receptor-associated factor 6/transforming growth factor-β activated kinase 1/TGF-beta-activated kinase 1 binding protein 2) pathway [23,114]. This leads to subsequent nuclear translocation of nuclear factor-kappa B (NF-κB) transcription factor, resulting in the transcription of proinflammatory cytokines, such as TNFα, IL-6 and IL-1β (Figure 3b) [23,106].

Activation of the TLR4-induced NF-κB signalling pathway has also been associated with a decreased cardiomyocyte contractility [54]. The TLR4-mediated TRAF6/TAK1/TAB2 pathway is involved in the activation of several mitogen-activated protein kinases, namely extracellular signal-regulated kinase (ERK), c-Jun N-terminal kinase (JNK) and p38 mitogen-activated protein kinases (p38), which initiate the translocation of activator protein 1 (AP-1) into the nucleus and the consequent transcription of proinflammatory molecules [106]. TLR4 may, therefore, contribute to the myocardial injury observed in SARS-CoV-2-infected patients by increasing ACE2 expression in cardiomyocytes, pericytes and fibroblasts, inducing a hyperinflammatory status and/or promoting an important depression of cardiomyocyte contractility [23,54,115,116]. 

## 7. Myocarditis as a Consequence of mRNA COVID-19 Vaccination

Myocardial inflammation has also been reported after COVID-19 mRNA vaccination, particularly in young adult males, where the incidence rate of myocarditis after the second dose of COVID-19 mRNA vaccine was estimated to be approximately 12.6 cases per million doses [24,25]. The median time from second vaccination to symptom onset was 2 days, with 90% of myocarditis cases emerging within a week from the second dose [117]. Although the risk of myocarditis associated with SARS-CoV-2 infection is greater than that related to COVID-19 mRNA vaccines, with a 100-fold higher incidence following infection than that observed post-vaccination [22], understanding the exact pathophysiological mechanisms underpinning the development of this cardiovascular complication after COVID-19 mRNA vaccination would be beneficial for a better management of such a complication and for the identification of potential predisposing factors [118]. Autoimmunity mechanisms, molecular mimicry, cytokine storm and sex hormone differences represent the hypotheses proposed to justify the COVID-19 mRNA vaccination-induced myocardial inflammation [24,118]. This type of vaccine consists of mRNA molecules, characterised by a 5’ cap structure attached to a 5’ untranslated region (UTR), a SARS-CoV-2 spike protein coding sequence and a 3’ UTR terminated by a 3’ poly-A tail [119,120]. Once internalised into host cells, the endosome-entrapped mRNA is released into the cytosol, where it is translated by ribosomes into spike protein [121,122]. This newly formed antigenic protein is degraded by proteasomes into peptide epitopes, which are transferred into the endoplasmic reticulum (ER) to be incorporated into MHC class I [120]. The resulting MHC class I-antigenic peptide epitope complex is presented on the plasma membrane, where, once recognised by CD8^+^ T cells, can trigger an immune response [120,122]. The result of these events is the induction of an adaptive immune response able to recognise and neutralise the SARS-CoV-2 spike protein [122]. 

An important aspect of SARS-CoV-2 mRNA vaccines is the use of nucleoside-modified mRNA, which is crucial to minimise the innate immune response due to an indiscriminate activation of proinflammatory pathways [120,123,124]. Indeed, the replacement of proinflammatory nucleosides with chemically or naturally modified analogues prevents the activation of RNA-detecting TLRs and activation of type I interferon signalling, therefore, suppressing the recognition of such mRNA as pathogenic [124,125].

However, in some cases, an aberrant immune response to the mRNA vaccine may still occur, resulting, therefore, in exaggerated immunoinflammatory activity, which might have a role in COVID-19 mRNA vaccine-induced myocarditis [24,118]. According to this hypothesis, the mRNA molecules released in endosomes or cytosol from the vaccine, acting as a mimic of viral mRNA, might be recognised as PAMPs by TLRs (e.g., TLR7 and TLR8) prior to the translation, therefore, stimulating the production of type I interferons and proinflammatory cytokines, which, as mentioned above, may have a role in the development of myocardial inflammation [124,126]. Furthermore, increased levels of NK cells have been detected in a patient who developed myocarditis after COVID-19 mRNA vaccination, suggesting a role of these immune cells in mRNA vaccine-induced myocardial injury, even though is not clear if this increase is the cause of this cardiovascular complication or a response to limit such a pathological process [24,127,128]. 

Another possible pathophysiological mechanism underlying SARS-CoV-2 mRNA vaccine-related myocarditis could involve the production of cardiac autoantibodies directed against self-antigens, which may be essential for myocardial function (i.e., cardiac myosin) [24,66,118,129]. These have been observed in non-COVID-19-related myocarditis [13,66,68], and it remains possible that the resulting autoantibodies might cross react with cardiac α-myosin heavy chain (α-MHC) and β-myosin heavy chain (β-MHC), therefore, triggering an autoimmune response against these structural myocardial components, with the result of an impaired myocardial contraction and consequent cardiomyopathy [13,68,129]. In addition, it has been shown that autoantibodies against cardiac myosin can also affect cardiomyocyte function through a cross-interaction with β-adrenergic receptors on cardiac cells, as a consequence of molecular mimicry between these two cardiac antigens [130,131]. The cross-reaction between anti-cardiac myosin autoantibodies and β-adrenergic receptors, leading to the activation of cyclic adenosine monophosphate (cAMP)-dependent PKA (protein kinase A) [130,131], could contribute to cardiomyocyte apoptosis, cardiac remodelling and impaired myocardial function and contractility [132]. This cross-reactivity may play a role even in the pathogenesis of SARS-CoV-2 mRNA vaccine-induced myocarditis, reinforcing the hypothesis of an aberrant autoantibody-mediated immune response [13]. In the context of the cardiac autoantibody theory, the hypothesis that pre-existing autoimmune diseases might promote SARS-CoV-2 mRNA vaccine-induced myocardial injury has not been confirmed in patients [24,26]. Serological markers of autoimmune diseases (e.g., antinuclear antibody test and rheumatoid factor tests) were performed in individuals that developed myocarditis after receiving the SARS-CoV-2 mRNA vaccine, showing no current evidence of a link between myocardial damage and pre-existing autoimmune disorders [24,26].

Interestingly, SARS-CoV-2 mRNA vaccine-induced myocarditis may also be the result of an unintentional intravenous injection [133]. The comparison of the histopathologic features of cardiac tissue, serum levels and mRNA myocardial tissue expression of proinflammatory cytokines after intravenous or intramuscular injection of SARS-CoV-2 mRNA vaccine in mice revealed that, although a sustained increase in proinflammatory cytokines (i.e., IL-1β, IFN-β, IL-6 and TNF-α) in the group receiving intramuscular injection was observed, only the animals receiving the vaccine intravenously reported significant histopathological changes typical of myocarditis [133]. Compared with intramuscular administration, the intravenous route was also associated with increased cardiomyocyte degeneration and apoptosis, as well as myocardial infiltration of inflammatory cells and increased serum levels of troponin [133]. As a result of these findings in an animal model, a plausible correlation might exist between the accidental intravenous administration of SARS-CoV-2 mRNA vaccine and the development of myocardial injury [133]. 

Molecular mimicry between the viral spike protein and cardiac proteins represents another plausible pathogenetic event proposed to explain myocardial damage as a result of COVID-19 mRNA vaccination [118,128,134]. In this context, antibodies directed against SARS-CoV-2 spike protein may recognise and cross-react with structurally related cardiac contractile proteins, such as α-myosin heavy chain, triggering a non-specific innate immune reaction against these myocardial components, with the consequent development of myocarditis [24,134,135]. However, the investigation of plausible cross-reactivity between SARS-CoV-2 spike protein encoded in mRNA COVID-19 vaccines and auto-antigens associated with myocarditis resulted in no significant similarity between vaccine peptides and myocarditis-associated autoantigens, therefore, contradicting the hypothesis of a cross-reactive response as a mechanism responsible for myocardial inflammation induced by COVID-19 mRNA vaccination [136].

Finally, considering the higher incidence of COVID-19 mRNA vaccine-induced myocarditis in young adult males, a hormonal hypothesis has been proposed [24,118]. In this regard, the inhibitory effect on anti-inflammatory responses promoted by testosterone, as well as its effects on Th1 helper cells, may provide an additional cause of SARS-CoV-2 mRNA vaccine-induce myocarditis [24,118]. Indeed, sex hormone signalling has an important role in the modulation of the immunoinflammatory response and in the control of mitochondrial function, with consequential sex differences in the development of autoimmune myocarditis [137]. While high levels of oestrogens appear to have a cardioprotective effect by promoting mitochondrial fusion in cardiomyocytes and triggering the activation of both Th2 CD4^+^ T-cell and anti-inflammatory M2 macrophages, testosterone favours mitochondrial fission, leading to increased ROS production and the release of mitochondrial DAMPs [137]. The final result is the activation of nucleotide-binding and oligomerization domain (NOD) -, leucine-rich repeat (LRR) and pyrin domain-containing protein 3 (NLRP3) inflammasome, culminating in a proinflammatory immune response with the release of interleukin-1 beta (IL-1β) and interleukin-18 (IL-18) and the activation of both M1 macrophages and Th1 subset of CD4^+^ T cells [137,138]. Moreover, it has been observed that severe myocardial inflammation in male mice was associated with higher expression of TLR4, which might further contribute to increasing the levels of proinflammatory IL-1β and IL-18, in turn, responsible for cardiomyocyte apoptosis and contractile dysfunction [137,139]. Indeed, IL-1β has been shown to determine cardiomyocyte apoptosis through the activation of both caspase-independent pathways (via upregulation of endonuclease G) and caspase-dependent pathways (via the release of cytochrome C and subsequent activation of caspase 3), as well as via the inhibition of survivin, a member of the inhibitor of apoptosis protein family [140], while IL-18, by binding TLRs, triggers the MyD88 signalling axis, with the consequent activation of NF-κB and increased production of iNOS (inducible nitic oxide synthase), resulting in a reduced myocardial contractile function [141]. These sex differences in the modulation of inflammatory responses and mitochondrial dynamics might also have a role in the pathogenesis of SARS-CoV-2 mRNA vaccine-induced myocarditis, thus potentially explaining the higher incidence of this cardiovascular complication in the male population [118,128]. 

In conclusion, the link between the SARS-CoV-2 mRNA vaccine and myocardial injury has not been clearly identified. However, aberrant immunoinflammatory events, the development of cardiac autoantibodies and their cross-reaction with myocardial components, as well as unintentional intravenous injection and molecular mimicry between spike protein and cardiac contractile proteins, represent the most appealing mechanisms behind this cardiovascular manifestation induced by the SARS-CoV-2 mRNA vaccine. These events may be exacerbated by sex differences resulting in increased risk of myocardial impairment. 

## 8. Conclusions and Future Directions

Several cases of myocarditis have been reported in SARS-CoV-2-infected patients and have been implicated with a poorer prognosis and disease course. The pathophysiological processes responsible for this myocardial injury are not yet fully elucidated, making the management and the prevention of such cardiovascular complication difficult and limited. A major role seems to be played by ACE2, whose SARS-CoV-2-mediated downregulation has profound effects on the cardiovascular system and, specifically, on myocardial tissue. In addition to a role of ACE2, it is also likely that Toll-like receptors promote further myocardial inflammation in COVID-19 patients. Damage of cardiac muscle has also been reported after SARS-CoV-2 mRNA vaccinations and this may also involve the activation of Toll-like receptors. Future studies are, therefore, needed to fully identify the pathogenetic mechanisms of post-infection and post-mRNA vaccination myocardial inflammation. A better understanding of these events will be essential, not only for an adequate management of the disease but also to identify a correct preventive approach to limit its manifestation. 

## Abbreviations

ACE2	angiotensin-converting enzyme 2
ADAM-17	a disintegrin and metalloprotease 17
Ang (1-7)	angiotensin (1-7)
Ang (1-9)	angiotensin (1-9)
Ang II	angiotensin II
AP-1	activator protein 1
APCs	antigen-presenting cells
cAMP	cyclic adenosine monophosphate
CD4+	helper T cells
CD8+	cytotoxic T cells
COVID-19	coronavirus disease 2019
DAMPs	damage-associated molecular patterns
DCM	dilated cardiomyopathy
dsRNA	double stranded RNA
dUTP	deoxyuridine, triphosphate
ERK	extracellular signal-regulated kinase
IFNAR1	interferon α and β receptor subunit 1
IFNAR2	interferon α and β receptor subunit 2
IFNα	interferon alpha
IFNγ	interferon gamma
IL-1	interleukin 1
IL-12	interleukin 12
IL-18	interleukin 18
IL-1α	interleukin 1 alpha
IL-1β	interleukin 1 beta
IL-6	interleukin 6
iNOS	inducible nitric oxide synthase
ISGs	interferon-stimulated genes
IκB	inhibitor of nuclear factor kappa B
JAK	Janus kinase
JNK	c-Jun N-terminal kinase
LRR	leucine-rich repeat
MasR	Mas receptor
MERS-CoV	Middle East respiratory syndrome coronavirus
MHC class I	major histocompatibility complex class I
mRNA	messenger ribonucleic acid
MyD88	myeloid differentiation factor 88
NF-kB	nuclear factor kappa B
NK	natural killer
NLRP3	NOD-, LRR- and pyrin domain-containing protein 3
NOD	nucleotide-binding and oligomerization domain
p38	p38 mitogen-activated protein kinases
PAMPs	pathogen-associated molecular patterns
PKA	protein kinase A
RAAS	renin-angiotensin-aldosterone system
RBD	receptor-binding domain
ROS	reactive oxygen species
SARS-CoV-1	severe acute respiratory syndrome coronavirus 1
SARS-CoV-2	severe acute respiratory syndrome coronavirus 2
SCD	sudden cardiac death
SG	stress granule
ssRNA	single stranded RNA
STAT	signal transducer and activator of transcription
TAB2	TGF-beta activated kinase 1 binding protein 2
TAK1	transforming growth factor-β activated kinase 1
TIR	Toll/IL-1 receptor homologous domain
TLR	Toll-like receptor
TMPRSS2	transmembrane serine protease 2
TNFα	tumour necrosis factor alpha
TNFβ	tumour necrosis factor beta
TRAF6	TNF receptor associated factor 6
TRAM	TRIF-related adaptor molecule
TRIF	TIR-domain-containing adapter-inducing interferon β
TUNEL	terminal deozynucleotidyl transferase dUTP nick end labelling
UTR	untranslated region
α-MHC	α-myosin heavy chain
β-MHC	β-myosin heavy chain

## References

[1] Sagar S., Liu P.P., Cooper L.T. (2012). Myocarditis. Lancet.

[2] Richardson P., McKenna W., Bristow M., Maisch B., Mautner B., O’Connell J., Olsen E., Thiene G., Goodwin J., Gyarfas I. (1996). Report of the 1995 World Health Organization/International Society and Federation of Cardiology Task Force on the Definition and Classification of cardiomyopathies. Circulation.

[3] Caforio A.L., Pankuweit S., Arbustini E., Basso C., Gimeno-Blanes J., Felix S.B., Fu M., Heliö T., Heymans S., Jahns R. (2013). Current state of knowledge on aetiology, diagnosis, management, and therapy of myocarditis: A position statement of the European Society of Cardiology Working Group on Myocardial and Pericardial Diseases. Eur. Heart J..

[4] Dennert R., Crijns H.J., Heymans S. (2008). Acute viral myocarditis. Eur. Heart J..

[5] Leone O., Pieroni M., Rapezzi C., Olivotto I. (2019). The spectrum of myocarditis: From pathology to the clinics. Virchows Arch..

[6] Lynge T.H., Nielsen T.S., Gregers Winkel B., Tfelt-Hansen J., Banner J. (2019). Sudden cardiac death caused by myocarditis in persons aged 1–49 years: A nationwide study of 14,294 deaths in Denmark. Forensic Sci. Res..

[7] Halle M., Binzenhöfer L., Mahrholdt H., Johannes Schindler M., Esefeld K., Tschöpe C. (2020). Myocarditis in athletes: A clinical perspective. Eur. J. Prev. Cardiol..

[8] Olejniczak M., Schwartz M., Webber E., Shaffer A., Perry T.E. (2020). Viral Myocarditis-Incidence, Diagnosis and Management. J. Cardiothorac. Vasc. Anesth..

[9] Fung G., Luo H., Qiu Y., Yang D., McManus B. (2016). Myocarditis. Circ. Res..

[10] Basso C. (2022). Myocarditis. New Engl. J. Med..

[11] Cooper L.T. (2009). Myocarditis. New Engl. J. Med..

[12] Esfandiarei M., McManus B.M. (2008). Molecular Biology and Pathogenesis of Viral Myocarditis. Annu. Rev. Pathol. Mech. Dis..

[13] Tschöpe C., Ammirati E., Bozkurt B., Caforio A.L.P., Cooper L.T., Felix S.B., Hare J.M., Heidecker B., Heymans S., Hübner N. (2021). Myocarditis and inflammatory cardiomyopathy: Current evidence and future directions. Nat. Rev. Cardiol..

[14] Siripanthong B., Nazarian S., Muser D., Deo R., Santangeli P., Khanji M.Y., Cooper L.T., Chahal C.A.A. (2020). Recognizing COVID-19-related myocarditis: The possible pathophysiology and proposed guideline for diagnosis and management. Heart Rhythm.

[15] Patone M., Mei X.W., Handunnetthi L., Dixon S., Zaccardi F., Shankar-Hari M., Watkinson P., Khunti K., Harnden A., Coupland C.A.C. (2022). Risks of myocarditis, pericarditis, and cardiac arrhythmias associated with COVID-19 vaccination or SARS-CoV-2 infection. Nat. Med..

[16] Ali M., Shiwani H.A., Elfaki M.Y., Hamid M., Pharithi R., Kamgang R., Egom C.B., Oyono J.L.E., Egom E.E.-A. (2022). COVID-19 and myocarditis: A review of literature. Egypt. Heart J..

[17] Alhogbani T. (2016). Acute myocarditis associated with novel Middle east respiratory syndrome coronavirus. Ann. Saudi Med..

[18] Lombardi A.F., Afsahi A.M., Gupta A., Gholamrezanezhad A. (2021). Severe acute respiratory syndrome (SARS), Middle East respiratory syndrome (MERS), influenza, and COVID-19, beyond the lungs: A review article. Radiol. Med..

[19] Van Linthout S., Klingel K., Tschöpe C. (2020). SARS-CoV-2-related myocarditis-like syndromes Shakespeare’s question: What’s in a name?. Eur. J. Heart Fail..

[20] Oudit G.Y., Kassiri Z., Jiang C., Liu P.P., Poutanen S.M., Penninger J.M., Butany J. (2009). SARS-coronavirus modulation of myocardial ACE2 expression and inflammation in patients with SARS. Eur. J. Clin. Investig..

[21] Babapoor-Farrokhran S., Gill D., Walker J., Rasekhi R.T., Bozorgnia B., Amanullah A. (2020). Myocardial injury and COVID-19: Possible mechanisms. Life Sci..

[22] Maiese A., Frati P., Del Duca F., Santoro P., Manetti A.C., La Russa R., Di Paolo M., Turillazzi E., Fineschi V. (2021). Myocardial Pathology in COVID-19-Associated Cardiac Injury: A Systematic Review. Diagnostics.

[23] Aboudounya M.M., Heads R.J. (2021). COVID-19 and Toll-Like Receptor 4 (TLR4): SARS-CoV-2 May Bind and Activate TLR4 to Increase ACE2 Expression, Facilitating Entry and Causing Hyperinflammation. Mediat. Inflamm..

[24] Bozkurt B., Kamat I., Hotez P.J. (2021). Myocarditis With COVID-19 mRNA Vaccines. Circulation.

[25] Witberg G., Barda N., Hoss S., Richter I., Wiessman M., Aviv Y., Grinberg T., Auster O., Dagan N., Balicer R.D. (2021). Myocarditis after Covid-19 Vaccination in a Large Health Care Organization. New Engl. J. Med..

[26] Abu Mouch S., Roguin A., Hellou E., Ishai A., Shoshan U., Mahamid L., Zoabi M., Aisman M., Goldschmid N., Berar Yanay N. (2021). Myocarditis following COVID-19 mRNA vaccination. Vaccine.

[27] Jackson C.B., Farzan M., Chen B., Choe H. (2022). Mechanisms of SARS-CoV-2 entry into cells. Nat. Rev. Mol. Cell Biol..

[28] Tikellis C., Thomas M.C. (2012). Angiotensin-Converting Enzyme 2 (ACE2) Is a Key Modulator of the Renin Angiotensin System in Health and Disease. Int. J. Pept..

[29] Bian J., Li Z. (2021). Angiotensin-converting enzyme 2 (ACE2): SARS-CoV-2 receptor and RAS modulator. Acta Pharm. Sin. B.

[30] Kawasaki T., Kawai T. (2014). Toll-Like Receptor Signaling Pathways. Front. Immunol..

[31] Frantz S., Ertl G., Bauersachs J. (2007). Mechanisms of disease: Toll-like receptors in cardiovascular disease. Nat. Clin. Pr. Cardiovasc. Med..

[32] Vallejo J.G. (2011). Role of toll-like receptors in cardiovascular diseases. Clin. Sci..

[33] Favere K., Bosman M., Klingel K., Heymans S., Van Linthout S., Delputte P.L., De Sutter J., Heidbuchel H., Guns P.J. (2021). Toll-Like Receptors: Are They Taking a Toll on the Heart in Viral Myocarditis?. Viruses.

[34] Becher P.M., Hinrichs S., Fluschnik N., Hennigs J.K., Klingel K., Blankenberg S., Westermann D., Lindner D. (2018). Role of Toll-like receptors and interferon regulatory factors in different experimental heart failure models of diverse etiology: IRF7 as novel cardiovascular stress-inducible factor. PLoS ONE.

[35] Magadum A., Kishore R. (2020). Cardiovascular Manifestations of COVID-19 Infection. Cells.

[36] Zhong J., Basu R., Guo D., Chow F.L., Byrns S., Schuster M., Loibner H., Wang X.-h., Penninger J.M., Kassiri Z. (2010). Angiotensin-Converting Enzyme 2 Suppresses Pathological Hypertrophy, Myocardial Fibrosis, and Cardiac Dysfunction. Circulation.

[37] Bader M., Turner A.J., Alenina N. (2020). ACE2, a multifunctional protein—From cardiovascular regulation to COVID-19. Clin. Sci..

[38] Zamorano Cuervo N., Grandvaux N. (2020). ACE2: Evidence of role as entry receptor for SARS-CoV-2 and implications in comorbidities. Elife.

[39] Turner A.J. (2015). ACE2 Cell Biology, Regulation, and Physiological Functions. Prot. Arm Renin Angiotensin Syst. (RAS).

[40] Samavati L., Uhal B.D. (2020). ACE2, Much More Than Just a Receptor for SARS-COV-2. Front. Cell. Infect. Microbiol..

[41] Hu Y., Liu L., Lu X. (2021). Regulation of Angiotensin-Converting Enzyme 2: A Potential Target to Prevent COVID-19?. Front. Endocrinol..

[42] Sampaio W.O., Souza dos Santos R.A., Faria-Silva R., da Mata Machado L.T., Schiffrin E.L., Touyz R.M. (2007). Angiotensin-(1-7) through receptor Mas mediates endothelial nitric oxide synthase activation via Akt-dependent pathways. Hypertension.

[43] Cooper S.L., Boyle E., Jefferson S.R., Heslop C.R.A., Mohan P., Mohanraj G.G.J., Sidow H.A., Tan R.C.P., Hill S.J., Woolard J. (2021). Role of the Renin-Angiotensin-Aldosterone and Kinin-Kallikrein Systems in the Cardiovascular Complications of COVID-19 and Long COVID. Int. J. Mol. Sci..

[44] Wang J., He W., Guo L., Zhang Y., Li H., Han S., Shen D. (2017). The ACE2-Ang (1–7)-Mas receptor axis attenuates cardiac remodeling and fibrosis in post-myocardial infarction. Mol. Med. Rep..

[45] Rabelo L.A., Todiras M., Nunes-Souza V., Qadri F., Szijártó I.A., Gollasch M., Penninger J.M., Bader M., Santos R.A., Alenina N. (2016). Genetic Deletion of ACE2 Induces Vascular Dysfunction in C57BL/6 Mice: Role of Nitric Oxide Imbalance and Oxidative Stress. PLoS ONE.

[46] Aleksova A., Gagno G., Sinagra G., Beltrami A.P., Janjusevic M., Ippolito G., Zumla A., Fluca A.L., Ferro F. (2021). Effects of SARS-CoV-2 on Cardiovascular System: The Dual Role of Angiotensin-Converting Enzyme 2 (ACE2) as the Virus Receptor and Homeostasis Regulator-Review. Int. J. Mol. Sci..

[47] Lovren F., Pan Y., Quan A., Teoh H., Wang G., Shukla P.C., Levitt K.S., Oudit G.Y., Al-Omran M., Stewart D.J. (2008). converting enzyme-2 confers endothelial protection and attenuates atherosclerosis. Am. J. Physiol. Heart Circ. Physiol..

[48] Hofmann U., Ertl G., Frantz S. (2011). Toll-like receptors as potential therapeutic targets in cardiac dysfunction. Expert Opin. Ther. Targets.

[49] Kumar H., Kawai T., Akira S. (2009). Pathogen recognition in the innate immune response. Biochem. J..

[50] El-Zayat S.R., Sibaii H., Mannaa F.A. (2019). Toll-like receptors activation, signaling, and targeting: An overview. Bull. Natl. Res. Cent..

[51] Sameer A.S., Nissar S. (2021). Toll-Like Receptors (TLRs): Structure, Functions, Signaling, and Role of Their Polymorphisms in Colorectal Cancer Susceptibility. Biomed. Res. Int..

[52] Kaisho T., Akira S. (2006). Toll-like receptor function and signaling. J. Allergy Clin. Immunol..

[53] Botos I., Segal D.M., Davies D.R. (2011). The structural biology of Toll-like receptors. Structure.

[54] Boyd J.H., Mathur S., Wang Y., Bateman R.M., Walley K.R. (2006). Toll-like receptor stimulation in cardiomyoctes decreases contractility and initiates an NF-kappaB dependent inflammatory response. Cardiovasc. Res..

[55] Kindermann I., Barth C., Mahfoud F., Ukena C., Lenski M., Yilmaz A., Klingel K., Kandolf R., Sechtem U., Cooper L.T. (2012). Update on Myocarditis. J. Am. Coll. Cardiol..

[56] Pollack A., Kontorovich A.R., Fuster V., Dec G.W. (2015). Viral myocarditis--diagnosis, treatment options, and current controversies. Nat. Rev. Cardiol..

[57] Liu P.P., Mason J.W. (2001). Advances in the Understanding of Myocarditis. Circulation.

[58] Liu P.P., Opavsky M.A. (2000). Viral Myocarditis. Circ. Res..

[59] Saraste A., Arola A., Vuorinen T., Kytö V., Kallajoki M., Pulkki K., Voipio-Pulkki L.M., Hyypiä T. (2003). Cardiomyocyte apoptosis in experimental coxsackievirus B3 myocarditis. Cardiovasc. Pathol..

[60] McManus B.M., Chow L.H., Wilson J.E., Anderson D.R., Gulizia J.M., Gauntt C.J., Klingel K.E., Beisel K.W., Kandolf R. (1993). Direct myocardial injury by enterovirus: A central role in the evolution of murine myocarditis. Clin. Immunol. Immunopathol..

[61] Badorff C., Lee G.H., Lamphear B.J., Martone M.E., Campbell K.P., Rhoads R.E., Knowlton K.U. (1999). Enteroviral protease 2A cleaves dystrophin: Evidence of cytoskeletal disruption in an acquired cardiomyopathy. Nat. Med..

[62] Maisch B., Ristić A.D., Hufnagel G., Pankuweit S. (2002). Pathophysiology of viral myocarditis: The role of humoral immune response. Cardiovasc. Pathol..

[63] Freeman G.L., Colston J.T., Zabalgoitia M., Chandrasekar B. (1998). Contractile depression and expression of proinflammatory cytokines and iNOS in viral myocarditis. Am. J. Physiol..

[64] Matsumori A., Yamada T., Suzuki H., Matoba Y., Sasayama S. (1994). Increased circulating cytokines in patients with myocarditis and cardiomyopathy. Br. Heart J..

[65] Massilamany C., Huber S.A., Cunningham M.W., Reddy J. (2014). Relevance of molecular mimicry in the mediation of infectious myocarditis. J. Cardiovasc. Transl. Res..

[66] Caforio A.L., Mahon N.J., Tona F., McKenna W.J. (2002). Circulating cardiac autoantibodies in dilated cardiomyopathy and myocarditis: Pathogenetic and clinical significance. Eur. J. Heart Fail..

[67] Wallukat G., Schimke I. (2014). Agonistic autoantibodies directed against G-protein-coupled receptors and their relationship to cardiovascular diseases. Semin. Immunopathol..

[68] Caforio A.L., Grazzini M., Mann J.M., Keeling P.J., Bottazzo G.F., McKenna W.J., Schiaffino S. (1992). Identification of alpha- and beta-cardiac myosin heavy chain isoforms as major autoantigens in dilated cardiomyopathy. Circulation.

[69] Wolff P.G., Kühl U., Schultheiss H.-P. (1989). Laminin distribution and autoantibodies to laminin in dilated cardiomyopathy and myocarditis. Am. Heart J..

[70] Limas C.J., Goldenberg I.F., Limas C. (1989). Autoantibodies against beta-adrenoceptors in human idiopathic dilated cardiomyopathy. Circ. Res..

[71] Limas C.J., Goldenberg I.F., Limas C. (1990). Influence of anti-beta-receptor antibodies on cardiac adenylate cyclase in patients with idiopathic dilated cardiomyopathy. Am. Heart J..

[72] Caforio A.L., Goldman J.H., Haven A.J., Baig K.M., Libera L.D., McKenna W.J. (1997). Circulating cardiac-specific autoantibodies as markers of autoimmunity in clinical and biopsy-proven myocarditis. The Myocarditis Treatment Trial Investigators. Eur. Heart J..

[73] Chen C., Zhou Y., Wang D.W. (2020). SARS-CoV-2: A potential novel etiology of fulminant myocarditis. Herz.

[74] Boehmer T.K., Kompaniyets L., Lavery A.M., Hsu J., Ko J.Y., Yusuf H., Romano S.D., Gundlapalli A.V., Oster M.E., Harris A.M. (2021). Association Between COVID-19 and Myocarditis Using Hospital-Based Administrative Data—United States, March 2020–January 2021. MMWR Morb. Mortal. Wkly. Rep..

[75] Guo T., Fan Y., Chen M., Wu X., Zhang L., He T., Wang H., Wan J., Wang X., Lu Z. (2020). Cardiovascular Implications of Fatal Outcomes of Patients With Coronavirus Disease 2019 (COVID-19). JAMA Cardiol..

[76] Dmytrenko O., Lavine K.J. (2022). Cardiovascular Tropism and Sequelae of SARS-CoV-2 Infection. Viruses.

[77] Gheblawi M., Wang K., Viveiros A., Nguyen Q., Zhong J.-C., Turner A.J., Raizada M.K., Grant M.B., Oudit G.Y. (2020). Angiotensin-Converting Enzyme 2: SARS-CoV-2 Receptor and Regulator of the Renin-Angiotensin System. Circ. Res..

[78] Lindner D., Fitzek A., Bräuninger H., Aleshcheva G., Edler C., Meissner K., Scherschel K., Kirchhof P., Escher F., Schultheiss H.-P. (2020). Association of Cardiac Infection With SARS-CoV-2 in Confirmed COVID-19 Autopsy Cases. JAMA Cardiol..

[79] Sharma R.K., Stevens B.R., Obukhov A.G., Grant M.B., Oudit G.Y., Li Q., Richards E.M., Pepine C.J., Raizada M.K. (2020). ACE2 (Angiotensin-Converting Enzyme 2) in Cardiopulmonary Diseases. Hypertension.

[80] Huang Y., Yang C., Xu X.-f., Xu W., Liu S.-w. (2020). Structural and functional properties of SARS-CoV-2 spike protein: Potential antivirus drug development for COVID-19. Acta Pharmacol. Sin..

[81] Shang J., Wan Y., Luo C., Ye G., Geng Q., Auerbach A., Li F. (2020). Cell entry mechanisms of SARS-CoV-2. Proc. Natl. Acad. Sci. USA.

[82] Lan J., Ge J., Yu J., Shan S., Zhou H., Fan S., Zhang Q., Shi X., Wang Q., Zhang L. (2020). Structure of the SARS-CoV-2 spike receptor-binding domain bound to the ACE2 receptor. Nature.

[83] Hoffmann M., Kleine-Weber H., Schroeder S., Krüger N., Herrler T., Erichsen S., Schiergens T.S., Herrler G., Wu N.-H., Nitsche A. (2020). SARS-CoV-2 Cell Entry Depends on ACE2 and TMPRSS2 and Is Blocked by a Clinically Proven Protease Inhibitor. Cell.

[84] Örd M., Faustova I., Loog M. (2020). The sequence at Spike S1/S2 site enables cleavage by furin and phospho-regulation in SARS-CoV2 but not in SARS-CoV1 or MERS-CoV. Sci. Rep..

[85] Zheng Z.-Q., Wang S.-Y., Xu Z.-S., Fu Y.-Z., Wang Y.-Y. (2021). SARS-CoV-2 nucleocapsid protein impairs stress granule formation to promote viral replication. Cell Discov..

[86] Zipeto D., Palmeira J.d.F., Argañaraz G.A., Argañaraz E.R. (2020). ACE2/ADAM17/TMPRSS2 Interplay May Be the Main Risk Factor for COVID-19. Front. Immunol..

[87] Heurich A., Hofmann-Winkler H., Gierer S., Liepold T., Jahn O., Pöhlmann S. (2014). TMPRSS2 and ADAM17 cleave ACE2 differentially and only proteolysis by TMPRSS2 augments entry driven by the severe acute respiratory syndrome coronavirus spike protein. J. Virol..

[88] Shulla A., Heald-Sargent T., Subramanya G., Zhao J., Perlman S., Gallagher T. (2011). A transmembrane serine protease is linked to the severe acute respiratory syndrome coronavirus receptor and activates virus entry. J. Virol..

[89] Wang K., Gheblawi M., Oudit G.Y. (2020). Angiotensin Converting Enzyme 2. Circulation.

[90] Palau V., Riera M., Soler M.J. (2020). ADAM17 inhibition may exert a protective effect on COVID-19. Nephrol. Dial. Transpl..

[91] Sharma R.K., Li J., Krishnan S., Richards E.M., Raizada M.K., Mohandas R. (2021). Angiotensin-converting enzyme 2 and COVID-19 in cardiorenal diseases. Clin. Sci..

[92] Rice G.I., Jones A.L., Grant P.J., Carter A.M., Turner A.J., Hooper N.M. (2006). Circulating activities of angiotensin-converting enzyme, its homolog, angiotensin-converting enzyme 2, and neprilysin in a family study. Hypertension.

[93] Almengló C., Couselo-Seijas M., Agra R.M., Varela-Román A., García-Acuña J.M., González-Peteiro M., González-Juanatey J.R., Eiras S., Álvarez E. (2021). Soluble angiotensin-converting enzyme levels in heart failure or acute coronary syndrome: Revisiting its modulation and prognosis value. J. Mol. Med..

[94] Wallentin L., Lindbäck J., Eriksson N., Hijazi Z., Eikelboom J.W., Ezekowitz M.D., Granger C.B., Lopes R.D., Yusuf S., Oldgren J. (2020). Angiotensin-converting enzyme 2 (ACE2) levels in relation to risk factors for COVID-19 in two large cohorts of patients with atrial fibrillation. Eur. Heart J..

[95] Yeung M.L., Teng J.L.L., Jia L., Zhang C., Huang C., Cai J.-P., Zhou R., Chan K.-H., Zhao H., Zhu L. (2021). Soluble ACE2-mediated cell entry of SARS-CoV-2 via interaction with proteins related to the renin-angiotensin system. Cell.

[96] Gonzalez S.M., Siddik A.B., Su R.-C. (2021). Regulated Intramembrane Proteolysis of ACE2: A Potential Mechanism Contributing to COVID-19 Pathogenesis?. Front. Immunol..

[97] Wang J., Zhao H., An Y. (2021). ACE2 Shedding and the Role in COVID-19. Front. Cell. Infect. Microbiol..

[98] Wu Z., Hu R., Zhang C., Ren W., Yu A., Zhou X. (2020). Elevation of plasma angiotensin II level is a potential pathogenesis for the critically ill COVID-19 patients. Crit. Care.

[99] Martens C.R., Accornero F. (2021). Viruses in the Heart: Direct and Indirect Routes to Myocarditis and Heart Failure. Viruses.

[100] Komarowska I., Coe D., Wang G., Haas R., Mauro C., Kishore M., Cooper D., Nadkarni S., Fu H., Steinbruchel D.A. (2015). Hepatocyte Growth Factor Receptor c-Met Instructs T Cell Cardiotropism and Promotes T Cell Migration to the Heart via Autocrine Chemokine Release. Immunity.

[101] Costela-Ruiz V.J., Illescas-Montes R., Puerta-Puerta J.M., Ruiz C., Melguizo-Rodríguez L. (2020). SARS-CoV-2 infection: The role of cytokines in COVID-19 disease. Cytokine Growth Factor Rev..

[102] Akhmerov A., Marbán E. (2020). COVID-19 and the Heart. Circ. Res..

[103] Aboudounya M.M., Holt M.R., Heads R.J. (2021). SARS-CoV-2 Spike S1 glycoprotein is a TLR4 agonist, upregulates ACE2 expression and induces pro-inflammatory M1 macrophage polarisation. BioRxiv.

[104] Choudhury A., Mukherjee S. (2020). In silico studies on the comparative characterization of the interactions of SARS-CoV-2 spike glycoprotein with ACE-2 receptor homologs and human TLRs. J. Med. Virol..

[105] Hirano T., Murakami M. (2020). COVID-19: A New Virus, but a Familiar Receptor and Cytokine Release Syndrome. Immunity.

[106] Mabrey F.L., Morrell E.D., Wurfel M.M. (2021). TLRs in COVID-19: How they drive immunopathology and the rationale for modulation. Innate Immun..

[107] Barhoum P., Chambrun M.P.d., Dorgham K., Kerneis M., Burrel S., Quentric P., Parizot C., Chommeloux J., Bréchot N., Moyon Q. (2022). Heterogeneity of Fulminant COVID-19--Related Myocarditis in Adults. J. Am. Coll. Cardiol..

[108] Anjum F.R., Anam S., Abbas G., Mahmood M.S., Rahman S.U., Goraya M.U., Abdullah R.M., Luqman M., Ali A., Akram M.K. (2021). Type I IFNs: A Blessing in Disguise or Partner in Crime in MERS-CoV-, SARS-CoV-, and SARS-CoV-2-Induced Pathology and Potential Use of Type I IFNs in Synergism with IFN-γ as a Novel Antiviral Approach Against COVID-19. Viral Immunol..

[109] Ziegler C.G.K., Allon S.J., Nyquist S.K., Mbano I.M., Miao V.N., Tzouanas C.N., Cao Y., Yousif A.S., Bals J., Hauser B.M. (2020). SARS-CoV-2 Receptor ACE2 Is an Interferon-Stimulated Gene in Human Airway Epithelial Cells and Is Detected in Specific Cell Subsets across Tissues. Cell.

[110] Onabajo O.O., Banday A.R., Stanifer M.L., Yan W., Obajemu A., Santer D.M., Florez-Vargas O., Piontkivska H., Vargas J.M., Ring T.J. (2020). and viruses induce a novel truncated ACE2 isoform and not the full-length SARS-CoV-2 receptor. Nat. Genet..

[111] Land W.G. (2021). Role of DAMPs in respiratory virus-induced acute respiratory distress syndrome—With a preliminary reference to SARS-CoV-2 pneumonia. Genes Immun..

[112] Chen L., Long X., Xu Q., Tan J., Wang G., Cao Y., Wei J., Luo H., Zhu H., Huang L. (2020). Elevated serum levels of S100A8/A9 and HMGB1 at hospital admission are correlated with inferior clinical outcomes in COVID-19 patients. Cell. Mol. Immunol..

[113] Scozzi D., Cano M., Ma L., Zhou D., Zhu J.H., O’Halloran J.A., Goss C., Rauseo A.M., Liu Z., Sahu S.K. (2021). Circulating mitochondrial DNA is an early indicator of severe illness and mortality from COVID-19. JCI Insight.

[114] Yang Y., Lv J., Jiang S., Ma Z., Wang D., Hu W., Deng C., Fan C., Di S., Sun Y. (2016). The emerging role of Toll-like receptor 4 in myocardial inflammation. Cell Death Dis..

[115] Vukusic K., Thorsell A., Muslimovic A., Jonsson M., Dellgren G., Lindahl A., Sandstedt J., Hammarsten O. (2022). Overexpression of the SARS-CoV-2 receptor angiotensin converting enzyme 2 in cardiomyocytes of failing hearts. Sci. Rep..

[116] Chen L., Li X., Chen M., Feng Y., Xiong C. (2020). The ACE2 expression in human heart indicates new potential mechanism of heart injury among patients infected with SARS-CoV-2. Cardiovasc. Res..

[117] Oster M.E., Shay D.K., Su J.R., Gee J., Creech C.B., Broder K.R., Edwards K., Soslow J.H., Dendy J.M., Schlaudecker E. (2022). Myocarditis Cases Reported After mRNA-Based COVID-19 Vaccination in the US From December 2020 to August 2021. JAMA.

[118] Heymans S., Cooper L.T. (2021). Myocarditis after COVID-19 mRNA vaccination: Clinical observations and potential mechanisms. Nat. Rev. Cardiol..

[119] Vogel A.B., Kanevsky I., Che Y., Swanson K.A., Muik A., Vormehr M., Kranz L.M., Walzer K.C., Hein S., Güler A. (2021). BNT162b vaccines protect rhesus macaques from SARS-CoV-2. Nature.

[120] Rijkers G.T., Weterings N., Obregon-Henao A., Lepolder M., Dutt T.S., van Overveld F.J., Henao-Tamayo M. (2021). Antigen Presentation of mRNA-Based and Virus-Vectored SARS-CoV-2 Vaccines. Vaccines.

[121] Wadhwa A., Aljabbari A., Lokras A., Foged C., Thakur A. (2020). Opportunities and Challenges in the Delivery of mRNA-Based Vaccines. Pharmaceutics.

[122] Park J.W., Lagniton P.N.P., Liu Y., Xu R.-H. (2021). mRNA vaccines for COVID-19: What, why and how. Int. J. Biol. Sci..

[123] Karikó K., Muramatsu H., Welsh F.A., Ludwig J., Kato H., Akira S., Weissman D. (2008). Incorporation of pseudouridine into mRNA yields superior nonimmunogenic vector with increased translational capacity and biological stability. Mol. Ther..

[124] Pardi N., Hogan M.J., Porter F.W., Weissman D. (2018). mRNA vaccines—A new era in vaccinology. Nat. Rev. Drug Discov..

[125] Pardi N., Hogan M.J., Naradikian M.S., Parkhouse K., Cain D.W., Jones L., Moody M.A., Verkerke H.P., Myles A., Willis E. (2018). Nucleoside-modified mRNA vaccines induce potent T follicular helper and germinal center B cell responses. J. Exp. Med..

[126] Zhang C., Maruggi G., Shan H., Li J. (2019). Advances in mRNA Vaccines for Infectious Diseases. Front. Immunol..

[127] Muthukumar A., Narasimhan M., Li Q.-Z., Mahimainathan L., Hitto I., Fuda F., Batra K., Jiang X., Zhu C., Schoggins J. (2021). In-Depth Evaluation of a Case of Presumed Myocarditis After the Second Dose of COVID-19 mRNA Vaccine. Circulation.

[128] Parra-Lucares A., Toro L., Weitz-Muñoz S., Ramos C. (2021). Cardiomyopathy Associated with Anti-SARS-CoV-2 Vaccination: What Do We Know?. Viruses.

[129] Vojdani A., Kharrazian D. (2020). Potential antigenic cross-reactivity between SARS-CoV-2 and human tissue with a possible link to an increase in autoimmune diseases. Clin. Immunol..

[130] Mascaro-Blanco A., Alvarez K., Yu X., Lindenfeld J., Olansky L., Lyons T., Duvall D., Heuser J.S., Gosmanova A., Rubenstein C.J. (2008). Consequences of unlocking the cardiac myosin molecule in human myocarditis and cardiomyopathies. Autoimmunity.

[131] Li Y., Heuser J.S., Cunningham L.C., Kosanke S.D., Cunningham M.W. (2006). Mimicry and Antibody-Mediated Cell Signaling in Autoimmune Myocarditis. J. Immunol..

[132] Rockman H.A., Koch W.J., Lefkowitz R.J. (2002). Seven-transmembrane-spanning receptors and heart function. Nature.

[133] Li C., Chen Y., Zhao Y., Lung D.C., Ye Z., Song W., Liu F.-F., Cai J.-P., Wong W.-M., Yip C.C.-Y. (2021). ntravenous injection of COVID-19 mRNA vaccine can induce acute myopericarditis in mouse model. Clin. Infect. Dis..

[134] Larson K.F., Ammirati E., Adler E.D., Cooper L.T., Hong K.N., Saponara G., Couri D., Cereda A., Procopio A., Cavalotti C. (2021). After BNT162b2 and mRNA-1273 Vaccination. Circulation.

[135] Segal Y., Shoenfeld Y. (2018). Vaccine-induced autoimmunity: The role of molecular mimicry and immune crossreaction. Cell. Mol. Immunol..

[136] Marrama D., Mahita J., Sette A., Peters B. (2022). Lack of evidence of significant homology of SARS-CoV-2 spike sequences to myocarditis-associated antigens. EBioMedicine.

[137] Di Florio D.N., Sin J., Coronado M.J., Atwal P.S., Fairweather D. (2020). Sex differences in inflammation, redox biology, mitochondria and autoimmunity. Redox Biol..

[138] Park S., Won J.-H., Hwang I., Hong S., Lee H.K., Yu J.-W. (2015). Defective mitochondrial fission augments NLRP3 inflammasome activation. Sci. Rep..

[139] Frisancho-Kiss S., Davis S.E., Nyland J.F., Frisancho J.A., Cihakova D., Barrett M.A., Rose N.R., Fairweather D. (2007). Cutting Edge: Cross-Regulation by TLR4 and T cell Ig Mucin-3 Determines Sex Differences in Inflammatory Heart Disease. J. Immunol..

[140] Shen Y., Qin J., Bu P. (2015). Pathways Involved in Interleukin-1β–Mediated Murine Cardiomyocyte Apoptosis. Tex. Heart Inst. J..

[141] O’Brien L.C., Mezzaroma E., Van Tassell B.W., Marchetti C., Carbone S., Abbate A., Toldo S. (2014). Interleukin-18 as a therapeutic target in acute myocardial infarction and heart failure. Mol. Med..

